# Henoch-Schönlein Purpura Following the First Dose of COVID-19 Viral Vector Vaccine: A Case Report

**DOI:** 10.3390/vaccines9101078

**Published:** 2021-09-25

**Authors:** Maria Maddalena Sirufo, Martina Raggiunti, Lina Maria Magnanimi, Lia Ginaldi, Massimo De Martinis

**Affiliations:** 1 Department of Life, Health and Environmental Sciences, University of L’Aquila, 67100 L’Aquila, Italy; maddalena.sirufo@gmail.com (M.M.S.); martinaraggiunti@libero.it (M.R.); linamaria.magnanimi01@icatt.it (L.M.M.); lia.ginaldi@cc.univaq.it (L.G.); 2 Allergy and Clinical Immunology Unit, AUSL 04 Teramo, 64100 Teramo, Italy

**Keywords:** Henoch-Schönlein purpura (HSP), IgA-mediated vasculitis, small vessels, palpable purpura, haematuria, COVID-19, ChAdOx1 nCoV-19 vaccine (AZD1222), Vaxzevria, haematuria, arthralgia, COVID-19 vaccine

## Abstract

A 76 year-old female came to our observation one week after the vaccination with ChAdOx1 nCoV-19 AZD1222 for the onset of purpuric rash on her gluteal and legs regions associated with coxalgia and episodes of macrohaematuria. Henoch-Schönlein purpura (HSP) was diagnosed on the basis of the revised criteria developed by the European League Against Rheumatism, the Paediatric Rheumatology International Trials Organization, and the Paediatric Rheumatology European Society (EULAR/PRINTO/PRES). HSP is a common IgA-mediated small vessel vasculitis, typical of childhood, that affects several systems and is characterized by a tetrad of dermatological, abdominal, joint, and renal manifestations. The Etiology of HSP is not completely understood, but it was observed following upper respiratory tract infections, medications, vaccinations, and malignancies. HSP has previously been reported following immunization with various vaccines, mostly within 12 weeks post, suggesting a possible correlation. To our knowledge, this is the first report of the possible association between COVID-19 ChAdOx1 nCoV-19 AZD1222 and the onset of HSP in a previously healthy woman. No similar cases were reported amongst 23.848 participants in the ChAdOx1 nCoV-19 AZD1222 trial.

## 1. Introduction

Growing apprehension about the risks of vaccination have been associated with the current global health situation. In the safety evaluation of a vaccine, it is difficult to take into account interindividual factors, such as the genetic predisposition to immune hyperstimulation and autoimmunity. Immunologic phenomena and autoimmune disorders are usually the result of an interplay of factors, such as genetic predisposition, epigenetic modification, and environmental triggers such as infections and drugs [1]. It is now established that viruses, as well as vaccines, can induce a hyperstimulation of the immune system and produce an anti-autoantigen response through mechanisms such as molecular mimicry, epitope spreading, bystander activation, and polyclonal spreading [2]. Swelling and pain at the injection site, fatigue, fever, chills, nausea, headache, muscle soreness, and joint pain are common side effects of almost all COVID-19 vaccines. In addition, specific vaccines show unique adverse events such as neutropenia with the AstraZeneca/Oxford vaccine [3].

Henoch–Schönlein purpura (HSP), also known as IgA vasculitis, is a disorder that causes the small blood vessels in skin, joints, intestines, and kidneys to become inflamed and bleed [4,5]. HSP mostly occurs in children and is less common in adults, with an estimated annual incidence of 0.8–1.8/100.000 adult patients [6]. The tetrad of palpable purpura, arthralgia/arthritis, gastrointestinal involvement, and glomerulonephritis characterizes HSP, but not all the features are necessarily present and other organs can also be involved [7]. The most striking feature of this form of vasculitis is a purplish rash, typically on the lower legs and buttocks. The diagnosis is of exclusion without specific diagnostic tests [8] and clinically based on the revised criteria developed by the European League Against Rheumatism, the Paediatric Rheumatology International Trials Organization and the Paediatric Rheumatology European Society (EULAR/PRINTO/PRES) in 2008, which includes mandatory and supportive criteria (Table 1) [9].

To date, the etiology of HSP is not completely understood and seems to be associated with genetic and environmental factors, infections, medications, vaccinations or malignancies [10]. Many types of viruses and bacteria are believed to be associated with the pathogenesis of IgA vasculitis, as they have the capacity to induce abnormal IgA autoimmune reactions in the host. The COVID-19 pandemic is recognized to cause vasculitis-like syndromes, and several reports have described COVID-19 associated IgA vasculitis [11]. HSP has been previously reported following immunization with various vaccines, mostly within 12 weeks post vaccination [5]. The ChAdOx1 nCoV-19 vaccine (AZD1222) was developed at Oxford University and consists of a replication-deficient chimpanzee adenoviral vector ChAdOx1, containing the SARS-CoV-2 structural surface glycoprotein antigen (spike protein; nCoV-19) gene [12]. The aim of this report is to highlight the possible association between COVID-19 ChAdOx1 nCoV-19 AZD1222 and first onset of HSP in a previously healthy adult. There were no cases reported amongst 23.848 participants in the ChAdOx1 nCoV-19 AZD1222 safety and immunogenicity trial [12].

## 2. Case Report

**Figure 1:**
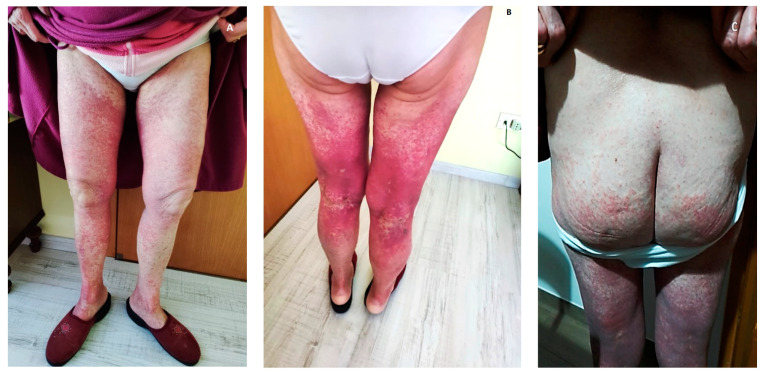
(**A**–**C**) Diffuse, non-whitening, maculopapular rash in both lower extremities.

We report the case of a 76 year-old female who came to our observation for the new onset of purpuric rash on her gluteal and legs regions associated with coxalgia and episodes of macrohaematuria. She had no significant previous medical history and received long-term supplementation with Calcifediol 1.5 mg/10 mL 20 drops/week per os for hypovitaminosis D. Seven days before, she received the first dose of the Vaxzevria (ChAdOx1 nCoV-19 AZD1222) vaccine. Skin examination showed diffuse, non-whitening, maculopapular rash not itchy in both lower extremities, extending from the soles to the thigh and involving the buttocks (Figure 1).

Her ankle joints were tender and painful and there was evidence of functional impotence of the limbs with difficulty in walking. Laboratory investigations revealed normal values for complete blood cell count, coagulation, kidney and liver function, autoimmune and neoplastic disease negative, but high values of CRP 40.85 mg/L and ESR 36 mm (Table 2). The urinalysis highlighted 72 red cells per high-power-field (HPF), and the urine culture report showed no evidence of growth. She did not have proteinuria. Instrumental examinations including chest radiograph, abdominal ultrasonography, and ecocolor-Doppler lower limb were normal. She did not undergo kidney and skin biopsy.

Positive criteria for vasculitic disease were the clinical picture of purpura, normal platelet count, and normal coagulation test results [13]. Furthermore, the patient fulfilled the EULAR/PRINTO/PRES criteria, as she had both the mandatory criterion, palpable purpura in the absence of thrombocytopaenia, and two of the supporting criteria, renal involvement in the form of haematuria and acute onset of arthralgia; the patient had the clinical signs and symptoms of HSP.

The trigger for the vasculitis was supposed to be ChAdOx1 nCoV-19 AZD1222 vaccination and, considering the renal and joint involvement, was proposed therapy with Paracetamol 1 g per os bis in die for seven days and Deflazacort 30 mg/die per os for ten days, then 15 mg/die per os for five days and, further, 7.5 mg/die per os for seven days and asked the patient to monitor signs and symptoms. After two weeks, signs resolved with normalization of laboratory parameters of ESR 10 mm, RCP 3.10, and urinalysis with no evidence of red cells. The arthritis also resolved. The patient, according to vaccination calendar, afterwards received the second dose, reporting only itching in the site of inoculum.

This work was conducted after receiving the patient’s informed consent to participate in the study and to publish this report and images, in compliance with the ethical standards in the field and the norms established by the Internal Review Board of University of L’Aquila (comitato etico di Ateneo D.R. n. 206/2013 and D.R. n. 46/2017).

## 3. Discussion and Conclusions

HSP has been observed in the setting of numerous infections and after the administration of several vaccines, including those against influenza, pneumococcal disease, rabies, hepatitis B virus, and meningococcal disease [14,15]. To date, several cases of HSP after SARS-CoV-2 infections are described in the literature [4,16,17,18,19,20,21,22,23,24,25,26,27,28]. Recently, Obeid et coll. reported a case of reactivation of IgA vasculitis occurring after COVID-19 vaccination and suggested a possible link between the reactivation of pre-existing IgA vasculitis observed after vaccination and the increase in anti-SARS-CoV-2 spike IgA. IgA vasculitis flares are reported following vaccinations and patients with IgA nephropathy show a stronger IgA response to intramuscular influenza vaccine respect to healthy controls. Furthermore, three cases of haematuria and IgA nephropathy flares following the second dose of mRNA COVID-19 vaccines have been reported in subjects with biopsy-proven IgA nephropathy, one patient following the BNT162b2 (BioNTech-Pfizer) vaccine, two of these after the mRNA-1273 vaccine [29].

To our knowledge, this is the first case described of HSP after the administration of a COVID-19 viral vector vaccine, which was likely the cause. Given the temporal association, this vaccine may have the potential to induce post-vaccination vasculitis, a rare adverse event. In the literature is described a case of HSV triggered by the Pfizer-BioNTech BNT16B2b2 mRNA vaccination [8]. It is possible that, after administration of the COVID-19 vaccine, immune complexes may form between vaccine antigens and native antibodies, thereby initiating a vasculitic process, as previously observed for other vaccinations [30]. IgA is driven in response to external stimuli and aberrant IgA responses underlie the pathogenesis of IgA vasculitis; IgA activates mannan-binding lectin and alternative complement pathways after creating IgA immunocomplexes and deposition on small vessels [11]. In the case reported, the problem of the booster dose was also addressed: should it be administered or not? Similar to other vaccines (e.g., influenza) [31], in the case of COVID-19 vaccination, cases are occurring of HSP after vaccination as well as pre-existing HSP exacerbated by the vaccine. Vaccinations could be associated with a variety of immunologic or immunologic-like adverse effects, but the precise pathogenetic mechanism remains unknown. Repeating the administration could potentially cause more severe immunologic reactions, but the antigen responsible for the reaction may not be the specific vaccine antigen (the spike protein in this case), furthermore, the product label with all vaccines and medications state that “hypersensitivity” to the product is a contraindication to its use.

There are currently no clear guidelines for the most appropriate management for patients with a history of vaccine-induced vasculitis and revaccination [32]. Using common sense and the standards of health professionals, after discussing with our patient the potential benefits and risks, it was decided to give the booster, and on this second occasion she had no reactions whatsoever. In the case described, we could not exclude the possibility that the occurrence of vasculitis following the COVID-19 vaccination was coincidental. As mass vaccination campaigns start worldwide, particularly in younger patients where HSP is more frequently observed, physicians should be aware of this complication, but should continue to encourage vaccination efforts given the well documented safety profile and efficacy of the ChAdOx1 nCoV-19 AZD1222 viral vector vaccine. Vaccination with COVID-19 vaccines is the only way today to eliminate, or at least control, COVID-19, and vaccine hesitancy is the potential nemesis [33]. Several rare but serious adverse reactions have been reported in the post marketing surveillance of COVID-19 vaccines. Monitoring the safety of COVID-19 vaccines is essential to improve safety profiles and enhance public trust. The correct information about these potential harms and the use of COVID-19 vaccines is essential [34]. People should be educated about their individual benefits and risks associated with COVID-19 vaccination, and we must ensure that vaccine recipients are aware of risks and that they should seek care if they experience concerning symptoms [3,35].

## Figures and Tables

**Table 1:** European League Against Rheumatism, the Paediatric Rheumatology International Trials Organization and the Paediatric Rheumatology European Society (EULAR/PRINTO/PRES) 2008 (7).

Diagnostic Criteria	Description
Mandatory criterion	Evidence of palpable purpura in the absence of thrombocytopaenia
Minimum 1 out of 4 supportive criteria	(1) Acute onset diffuse abdominal pain
	(2) Acute onset arthralgia or arthritis
	(3) Renal involvement in the form of proteinuria or haematuria
	(4) Histopathological evidence of leucocytoclastic vasculitis or proliferative glomerulonephritis with predominant IgA deposits

**Table 2:** Blood Tests.

Blood Tests	Value	Reference Ranges
Blood Cells Count		
Leukocyte	7.63 × 10^3^/µL	4.00–10.00
Neutrophils	5.42 × 10^3^/µL	2.00–7.00
Lymphocytes	1.65 × 10^3^/µL	1.00–4.00
Monocytes	0.24 × 10^3^/µL	0.20–1.00
Eosinophils	0.19 × 10^3^/µL	0.02–0.50
Basophils	0.01 × 10^3^/µL	0.00–0.20
Erythrocyte	4.75 × 10^6^/µL	3.8–4.8
Platelet	268 × 10^3^/µL	150–450
Erythrocyte sedimentation rate (ESR)	36 mm	1.0–15.0
C-reactive protein (CRP)	40.85 mg/dL	0.00–5.00
Kidney Function		
Creatinine	0.7 mg/dL	0.5–0.9
Liver Function		
Aspartate aminotransferase (AST)	23 U/L	4–32
Alanine aminotransferase (ALT)	19 U/L	4–32
Coagulation Test		
Prothrombin time (PT)	96% 11.9 s	70.0–130 10.0–14
International normalized ratio (INR)	10.3	0.80–1.30
Partial thromboplastin time (PTT)	25 s	23
Fibrinogen	280 mg/dL	200–430
Haptoglobin	120 mg/dL	50.0–220.0
Autoimmune Tests		
Antinuclear antibodies (ANA)	Negative	Negative
Extractable nuclear antigen (ENA) SCREENING (anti-Sm, RNP, Ro60, Ro62, SS-B, SCl-70, Jo-1)	<3.6 U/mL	<20
Antineutrophil cytoplasm antibodies (ANCA) directed against myeloperoxidase (MPO)	<3.20 U/mL	<20
ANCA-directed against proteinase-3 (PR3)	<2.30 U/mL	<20
Antiplatelet antibodies	Absent	Absent
Anticardiolipin IgG antibodies	0.40 U/mL	<7
Anticardiolipin IgM antibodies	2 U/mL	<7
Anti-β2-glycoprotein IgG antibodies	0.73 U/mL	<7
Anti-β2-glycoprotein IgM antibodies	0.21 U/mL	<7
Anti-double stranded DNA (Anti-dsDNA) antibodies	Negative	Negative
Immunoglobulins		
Cryoglobulin	Absent	Absent
Immunoglobulin G	1220 mg/dL	700.0–1600.0
Immunoglobulin A	325 mg/dL	70.0–400.0
Immunoglobulin M	107 mg/dL	40.0–230.0
